# SARS Control and Psychological Effects of Quarantine, Toronto, Canada

**DOI:** 10.3201/eid1102.040760

**Published:** 2005-02

**Authors:** Harry F. Hull

**Affiliations:** * Minnesota Department of Health, Minneapolis, Minnesota, USA

**Keywords:** SARS quarantine, psychological effects, letter

**To the Editor:** Hawryluck et al. (*1*) have published an interesting study that found that some persons subject to quarantine for severe acute respiratory distress syndrome (SARS) displayed symptoms of posttraumatic stress disorder and depression. They conclude that the psychological symptoms result from quarantine. I believe the study has serious flaws and that their conclusion is premature.

First, their study sampled 129 volunteers among the >15,000 persons subjected to quarantine. As acknowledged by the authors, persons with the most severe symptoms may be more likely to volunteer for the study, resulting in an overestimation of the frequency and severity of the symptoms. Second, more than two thirds of the participants were healthcare workers. Healthcare workers in Toronto who cared for SARS patients but were not subject to quarantine were experiencing extreme stress because they were working with a poorly understood infectious disease, wearing protective equipment for extended periods, and watching colleagues become ill and die while wondering if they themselves were the next victims. Most healthcare workers subject to quarantine in Toronto (including 34% of persons on work quarantine) likely cared for SARS patients and would have experienced stresses similar to those not quarantined. Third, 85% of the study participants wore masks at home, indicating that they were likely to have been symptomatic and subject to isolation rather than quarantine. Certainly symptomatic persons would be undergoing stress because of their concerns about SARS developing, the possibility of dying, and the potential for exposing others. Increasing levels of stress with increasing length of isolation found in the study may be due to more severe or prolonged symptoms rather than to isolation or quarantine per se.

Measuring the psychological effects of isolation and quarantine will require studies comparing psychological symptoms of healthcare workers subjected to quarantine with those who continued working, as well as studies comparing randomly selected persons subject to isolation with the general population living in the city during the outbreak.

In the final analysis, although isolation and quarantine are stressful, that is an insufficient reason to hesitate when these measures are indicated. One might wonder how stressed the participants would have been if SARS had developed and they infected their family members or friends. Regardless of whether isolation and quarantine induce posttraumatic stress disorder, public health officials must be cognizant of and prepared to supply appropriate emotional and social support to persons subject to isolation or quarantine.

## References

[1] Hawryluck L, Gold WL, Robinson S, Pogorski S, Galea S, Styra R. SARS control and psychological effects of quarantine, Toronto, Canada. Emerg Infect Dis. 2004;10:1206–12. 15324539 10.3201/eid1007.030703 PMC3323345

