# Supplementary material for: SARS Control and Psychological Effects of Quarantine, Toronto, Canada
Source: Emerg Infect Dis. 2005 Feb;11(2):354–5. doi: 10.3201/eid1102.040760 (PMC3320456; doi:10.3201/eid1102.040760)
Supplement: Related texts

# Reply: SARS Control and Psychological Effects of Quarantine, Toronto, Canada

**DOI:** 10.3201/eid1102.040978

**Authors:** Rima Styra, Laura Hawryluck, Wayne Gold

**Affiliations:** * University Health Network, Toronto, Ontario, Canada

**In Response:** Dr. Hull raises concerns regarding our study design and the conclusions that were drawn, believing the conclusions to be premature (*1*). To reiterate, we concluded that quarantine might result in considerable psychological distress in the forms of posttraumatic stress disorder (PTSD) and depressive symptoms, but we clearly qualify this conclusion by stating that the results of the study are hypothesis-generating and require further exploration.

Dr. Hull correctly writes that more than two thirds of the respondents to our survey were healthcare workers and assumes that healthcare workers in Toronto who cared for patients with severe acute respiratory syndrome (SARS) were extremely stressed. We agree with this statement not on the basis of data presented in this study, but rather on additional work that we have conducted on nonquarantined, uninfected healthcare workers treating patients with SARS in a hospital in Toronto (*2*). The implication of Dr. Hull's statement, however, is that being a healthcare worker in Toronto at the time of SARS, rather than being placed into quarantine, was responsible for the psychological distress that we measured. To dispute this, we found that healthcare worker status was not correlated with PTSD or depression symptoms, which indicates that respondents who were nonhealthcare workers experienced similar levels of distress as healthcare workers who responded. Furthermore, we found that longer durations of quarantine were associated with increased symptoms of PTSD, which indicates that the physical state of being in quarantine was at least in part responsible for the psychological distress.

Finally, Dr. Hull states that 85% of the study participants wore masks at home, which indicates that they were likely to have been symptomatic and subject to isolation rather than quarantine. This statement is incorrect. The respondents to this survey were asymptomatic, exposed persons who were placed into quarantine. Instructions to all quarantined persons, per public health guidelines (*3*), were to wear masks while in the presence of other household members, not because they were symptomatic, but rather because they may have been incubating SARS and had the potential to transmit infection to household contacts in the 24 hours before symptom onset.

Although the terms "isolation" and "quarantine" have often been used interchangeably, they actually represent distinct concepts (*4*). The strategies differ in that isolation applies to persons who are known to have an illness, and quarantine applies to those who have been exposed to a transmissible pathogen but who may or may not become ill. Quarantine directives for SARS included the adherence to home infection control measures, including wearing masks in the presence of other household members, not sharing utensils, and sleeping in separate quarters (*3*).

We agree with Dr. Hull's final statement that the psychological distress experienced by persons in quarantine is not a sufficient reason to refrain from invoking these measures when they are needed to control an outbreak. We did not arrive at this conclusion in our article. The goal of the study was to develop a benchmark for the possible distress associated with quarantine. While we felt that documenting the possible distress that may result from quarantine was important, it was not intended to negate the need to impose quarantine should it be required, but rather to determine the support measures that may be needed by quarantined persons. Public health officials must be cognizant of these needs and prepared to supply appropriate emotional and social support to persons in quarantine for such measures to succeed in halting the spread of disease.

## References

[1] Hull HF. SARS control and psychological effects of quarantine, Toronto, Canada [letter]. Emerg Infect Dis. 2005;11:353–4.1575934610.3201/eid1102.040760PMC3320456

[2] Gold WL, Hawryluck L, Robinson S, McGeer A, Fones C, Kennedy S, Post-traumatic stress disorder (PTSD) among healthcare workers (HCW) at a hospital treating patients with SARS [abstract]. In: Program and abstracts of the 43rd meeting of the Interscience Conference on Antimicrobial Agents and Chemotherapy (ICAAC); 2003 Sep 14–17; Chicago. Washington (DC): American Society for Microbiology; 2003.

[3] Svoboda T, Henry B, Shulman L, Kennedy E, Rea E, Ng W, Public health measures to control the spread of the severe acute respiratory syndrome during the outbreak in Toronto. N Engl J Med. 2004;350:2352–61. 10.1056/NEJMoa03211115175437

[4] Centers for Disease Control and Prevention. Public health guidance for community-level preparedness and response to severe acute respiratory syndrome (SARS) Version 2/3. 2004 Jul 20 [cited 2004 Sep 26]. Available from http://www.cdc.gov/ncidod/sars/guidance
